# Metatranscriptomics From a Small Aquatic System: Microeukaryotic Community Functions Through the Diurnal Cycle

**DOI:** 10.3389/fmicb.2020.01006

**Published:** 2020-05-25

**Authors:** Stephanie Trench-Fiol, Patrick Fink

**Affiliations:** ^1^Workgroup Aquatic Chemical Ecology, Institute for Zoology, University of Cologne, Cologne, Germany; ^2^Department of Aquatic Ecosystem Analysis and Management, Helmholtz Centre for Environmental Research – UFZ, Magdeburg, Germany; ^3^Department River Ecology, Helmholtz Centre for Environmental Research – UFZ, Magdeburg, Germany

**Keywords:** algae, gene expression, day/night cycle, metatranscriptome, microeukaryotes, ponds, RNA sequencing

## Abstract

Light is an important factor for the growth of planktonic organisms, and many of them depend on the diurnal light/dark cycle to regulate key metabolic processes. So far, most of the diel responses were only studied in single species or marine and large lake communities. Yet, we lack information on whether these processes are regulated similarly in small aquatic systems such as ponds. Here, we investigated the activity of a microeukaryotic community from a temperate, small freshwater pond in response to the diurnal cycle. For this, we took samples at midday and night during the Central European summer. We extracted pigments and RNA from samples and the sequencing of eukaryotic transcripts allowed us to obtain day and night metatranscriptomes. Differentially expressed transcripts primarily corresponded to photosynthesis-related and translational processes, and were found to be upregulated at midday with high light conditions compared to darkness. Unique gene ontology classes were found at each respective condition. During the day, ontology classes including photoreception for photosynthesis, defense, and stress mechanisms dominated, while motility, ribosomal assembly and other large, energy-consuming processes were restricted to the night. *Euglenophyta* and *Chlorophyta* dominated the active phototrophic community, as shown by the pigment composition analysis. Regarding the gene expression patterns, we could confirm that the pond community appears to follow similar diurnal dynamics as those described for larger aquatic ecosystems. Overall, combining pigment analyses, metatranscriptomics, and data on physicochemical factors yielded considerably more insight into the metabolic processes performed by the microeukaryotic community of a small freshwater ecosystem.

## Introduction

Light plays an important role in structuring microbial communities. For photosynthetic organisms like phytoplankton, light is the source of energy and is therefore crucial for primary production in aquatic systems. Many organisms have the ability to synchronize their metabolism to the light periods and organize cellular processes in response to daily light fluctuations (reviewed in Roenneberg and Merrow, 2005). This rhythmic response of cellular metabolisms to light/dark cycles is known as the circadian clock and in the case of microalgae, the molecular mechanisms have been reported for some model organisms (Mittag et al., 2005; Moulager et al., 2007; Monnier et al., 2010; O-Neill et al., 2011). Gene expression approaches have been key to elucidate the responses of these model organisms to nutrient and light availability (Bailleul et al., 2010; Gifford et al., 2013; Allorent et al., 2016; Jaubert et al., 2017). However, although crucial for the identification of different functions at gene or protein level and the understanding of main biological processes, single-organism studies under laboratory conditions hardly picture natural communities that experience unsteady environmental conditions. Thus, metatranscriptomics (transcript sequencing from the whole community) is a more suitable tool to provide a snapshot of the main active organisms and the predominant activities performed by a specific community in response to changing conditions (Vila-Costa et al., 2010; Gifford et al., 2011; Marchetti et al., 2012; Grossmann et al., 2016; Bižic-Ionescu et al., 2018). Nevertheless, metatranscriptomics alone is not sufficient to determine the taxonomic composition of an active community and demands complementary approaches (Shakya et al., 2019). For phytoplankton communities, the analysis of group-specific accessory pigment patterns provides a fast method that is frequently used to identify phytoplankton functional taxonomic composition (Mackey et al., 1996; Sarmento and Descy, 2008; Schlüter et al., 2016).

Previous metatranscriptomic research on the response of microbial communities to the natural day-night cycle has been mainly performed during summer when a diurnal change of temperature, light, and irradiance is pronounced and high biomass of phototrophic microorganism is present. These studies have shown expression patterns that link cellular processes to different times of the day, and that have been related to light availability. Photosynthesis and transporters-related transcripts, as well as transcripts involved in energy production and stress mechanisms, were preferentially expressed during the day. Whereas, carbon fixation, carbohydrates and amino acid synthesis, and cell division are processes that mainly occur during the night (Poretsky et al., 2009a; Vila-Costa et al., 2013; Aylward et al., 2015; Linz et al., 2019). Yet, most of these studies were done with communities from the open oceans and large lakes, while smaller aquatic ecosystems were so far overlooked.

Such small aquatic systems like ponds are particularly suitable to study the response of natural communities to environmental changes, as isolated systems are more susceptible to small scale environmental variations, these communities are more responsive to dynamic stimuli (Simon et al., 2015; Verbeek et al., 2018). It is of interest, as the global temperatures increase, to study pond systems that are subject to more frequent drought events, and to understand how their planktonic communities will be affected and respond by developing or not an adaptation to drastic environmental changes (Simon et al., 2016).

We hence aimed the present study to investigate the activity of a microeukaryotic community from a small freshwater pond during day and night. We expected the phototrophic fraction of the pond community to be more affected by light changes throughout the day, than the heterotrophic fraction. By measuring community-wide gene expression, we wanted to investigate whether the pond community responds to the diurnal cycle in the same way that has been reported for larger aquatic systems. To do so, we obtained metatranscriptomes together with pigment composition profiles from replicate day and night summer samplings over a month.

## Materials and Methods

### Sampling

Water samples were taken from a small (2 m× 2 m, ∼1 m depth), artificial pond located in the Botanical Gardens of the University of Cologne, Germany (50°55′31.0″N 6°56′09.4″E) during the summer of 2017 (June–July, with a light-dark ratio of ∼16:8 h). Samples were taken on 4 different days within a month. These 4 days were considered as four replicates for each daytime. Daytime samplings were conducted at midday, representing the maximum daily irradiance (∼7 h after sunrise). Nighttime samplings were conducted at 4:30 am with no measurable irradiance (∼7 h after sunset). At each sampling point, we determined the following physicochemical parameters *in situ*: temperature, pH (pH meter Vario. WTW, Germany), photosynthetically active radiation (PAR) in the air (at the pond’s surface) and in the water directly beneath the surface (Underwater Spherical Quantum Sensor LI-193. LI-COR Biosciences, United States), dissolved oxygen (DO, HQd portable meter with an optical DO sensor, Hach), and conductivity (Conductivity hand-held meter LF330, WTW, Germany).

### Sample Processing

Upon sampling, the collected water was immediately pre-filtered through a 30 μm mesh, before 500 mL of pre-filtered water was pumped through Sterivex^TM^ cartridges (0.2 μm) via a peristaltic pump with sterile Tygon tubing. Up to 2 mL of RNAlater (Qiagen) were added to each cartridge to preserve the samples before storage at −80°C until RNA extraction.

For pigment analyses, replicates of 200 mL of the pre-filtered water were filtered at low light conditions through GF/F filters ∅25 mm with a vacuum pump. Filters were wrapped in aluminum foil and stored at −20°C until acetone extraction and analysis on HPLC according to Ilic (2019). Briefly, filters were placed on vials with 3.5 ml 100% acetone (HPLC grade), sonicated for 2 min and then put on ice for 1 min, these two steps were repeated five times. The vials were then kept at 4°C overnight. On the next day, filters were removed and the extracts were centrifuged at 4500 × *g* for 15 min and 1 ml of the extracts were used to evaporate to dryness under nitrogen gas. 100 ng of trans-β-apo-8′-carotenal (Sigma Aldrich) was added to the extracts prior to evaporation and used as an internal standard. The evaporated extracts were resuspended in 100 μl of acetone and were transferred to HPLC vials, 25 μl from each sample were injected into the HPLC (Shimadzu Prominence system with a binary pump) onto a Spherisorb ODS2 column (stationary octadecyl-phase C_18_). The solvent A was methanol: ammonium acetate (1 M): acetonitrile (50:20:30 v/v), and solvent B was acetonitrile: ethyl acetate (50:50 v/v). The solvents gradient started at A: 90% and B: 10%, after 2 min A: 90% and B: 10%, after 26 min A: 40% and B: 60%, after 28 min A: 10% and B: 90%, after 30 min A: 10% and B: 90%. Pigments were identified via comparisons of retention times with those of pure standards DHI Water (Høersholm, Denmark). The pigment content from each sample was used to estimate the contribution of different phytoplankton groups to the total chlorophyll *a*, using the software CHEMTAX (Mackey et al., 1996), and initial matrices with pigment:chl-*a* ratios previously described for freshwater systems (Sarmento and Descy, 2008; Schlüter et al., 2016). A total of 60 randomized ratio matrices were constructed from the initial ratios for each dataset, and the program was run 60 times. The ratio limits were set to 500. The 10% of matrices with the lowest residual root mean square (RMS) were averaged and used as new input ratio matrices.

For the analysis of the particulate carbon and nitrogen fractions of the pond seston, volumes of 200 mL were filtered through GF/F filters ∅25 mm with a vacuum pump before drying them for 24 h at 60°C. Dried filters were subsequently wrapped into tin capsules and analyzed on a Thermo Flash EA2000 Analyzer (Schwerte, Germany). Soluble reactive phosphorus (SRP) was determined spectrophotometrically (DR5000 UV-Vis spectrophotometer. Hach, Germany) using the ascorbic acid molybdenum-blue method (Greenberg et al., 1985). For this, 100 mL of each water sample were filtered through GF/F filters and the filtrate collected in an acid-washed flask (5% sulfuric acid), measurements were done in triplicates were used each time. For the dissolved organic carbon (DOC) estimation, 100 mL of water were filtered through a 0.2 μm cellulose membrane filter and was kept in darkness at 4°C until analysis. The measurements were conducted using the Aqualog fluorometer (HORIBA) at excitation wavelengths 250–600 nm and emission wavelengths 250–620 nm, the index calculation was done with the R package staRdom (Pucher et al., 2019). All physicochemical data were tested for normality with the Shapiro-Wilk tests. *T*-tests or Mann-Whitney rank-sum tests were used to determine significant differences between day and night samples for all parameters. For the relative and absolute pigment composition, two-way ANOVAs (day/night and phyla) were performed, with subsequent pairwise multiple comparisons (Tukey test) for the different sampling dates and phyla. Statistical analyses were performed on R, version 3.6.1 (R Core Team, 2019).

### RNA Extraction and Sequencing

Sterivex^TM^ cartridges were thawed on ice, opened and processed as described by Cruaud et al. (2017). Membrane filters were cut into strips to fit on sterile screw-cap 2 mL tubes (CK14 tubes) for the RNA extraction with ReliaPrep kit (Promega), including DNase treatment. The fibrous tissue protocol was applied according to the manufacturer’s specification, using the lower amounts of reagents of the protocol, and an initial mechanical disruption of the samples using 0.65 g of 1.4 mm ceramic (zirconium) beads. Total RNA quality (A260/A280 and A260/A230 ratios) were assessed with a Nanodrop photometer, and sample concentrations were measured using the Qubit^TM^ fluorimeter’s RNA HS Assay Kit (Invitrogen). RNA integrity (RIN) of the total RNA samples (∼7) was checked with a Bioanalyzer (Agilent Technologies). Samples were poly-A selected for mRNA before cDNA libraries were prepared using the TruSeq^TM^ stranded kit, and 75 bp paired-end sequencing was performed in the Cologne Center for Genomics using the Illumina HiSeq 4000 platform.

### Metatranscriptome Analyses

Quality trimming of the sequences (all Phred scores > 30) was done with Trimmomatic 0.36 (Bolger et al., 2014) with parameters LEADING:5 TRAILING:5 MINLEN:70 (Phred 33) and remaining rRNA sequences were removed using Sortmerna 2.1 (Kopylova et al., 2012). Merging of paired ends sequences done with FLASH (Magoè and Salzberg, 2011) before the assembly gave only ∼30% of overlapping sequences that could be merged from the total raw sequences. Therefore, all sequences were used to perform *de novo* assemblies with the software Trinity version 2.5.1 (Haas et al., 2013). Raw sequences, samples and replicates individually, were aligned to the reference assemblies using Bowtie2, and then RSEM (Li and Dewey, 2014) was used to estimate transcripts abundances and create gene and isoform count matrices including all replicates and conditions. These matrices were first used to check between replicates and samples correlation, and then to perform differential expression analyses comparing the day and night samples. Differential expression analysis on the transcript level was done with the edgeR package (Robinson et al., 2010) and TMM normalization method (Robinson and Oshlack, 2010), using the default adjusted *p*-value cutoff of 0.001 for false discovery rate (FDR, Benjamini and Hochberg, 1995). The sequences from the complete day and night assemblies and the differentially expressed transcripts were annotated separately following the Trinotate pipeline (Bryant et al., 2017). This pipeline includes the prediction of coding regions on transcripts (TransDecoder)^1^, and uses Blastx and Blastp against the Swissprot database to get annotations from both transcripts and predicted proteins. For protein domain prediction, HMMR is used with the PFAM database. Taxonomical assignments, gene ontologies, and KEGG orthology were assigned from these annotations. Gene Ontology enrichment analysis for differentially expressed transcripts was performed using the R package GOseq (Young et al., 2010), the significance for GO categories (*p* < 0.05) was calculated after random resampling to generate a null distribution for each category amongst the over-represented DE transcripts. To find the over enriched GO categories, a 0.05 FDR was used.

The data from gene expression and gene ontology analyses is available on the Github repository https://github.com/sjtf89/Cologne_pond. The raw sequences are deposited in the sequence read archive (SRA) database from NCBI under the BioProject PRJNA596111.

## Results

### Environmental Parameters, Nutrient Content, and Pigment Composition

**FIGURE 1:**
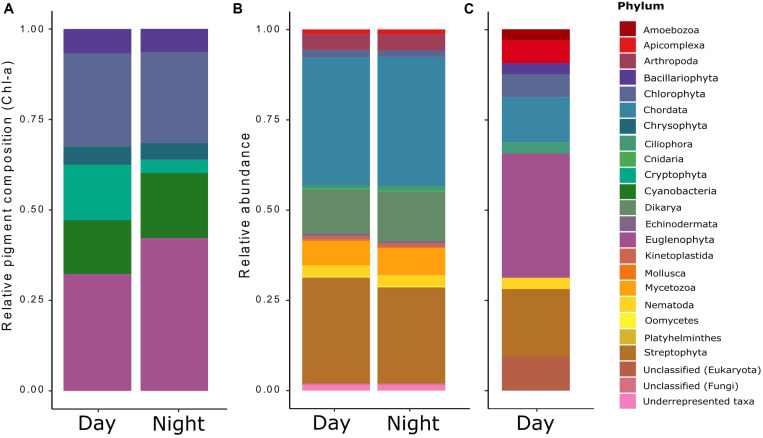
Relative taxonomic composition of the pond’s microeukaryotic community as derived from three distinct methods/data sets: **(A)** the contribution of phototrophic organisms (microalgae and cyanobacteria) derived from HPLC analyses of accessory photopigments (relative to chlorophyll *a*) via the matrix-factorization algorithm CHEMTAX (Mackey et al., 1996), **(B)** the general functional annotation of all eukaryotic transcripts obtained by RNA-Seq and determined via Trinotate; and **(C)** the functional annotation of the differentially expressed (DE) transcripts found for the RNA-Seq data.

The temperatures and PAR (air and water), and the dissolved oxygen measured *in situ* showed diel rhythmicity, with values that were significantly different for day and night, while this was not the case for pH and conductivity (Table 1). From the determined nutrients, only particulate organic carbon (POC) concentrations differed significantly between day and night samples (*p* < 0.05). Particulate organic nitrogen (PON) did not vary greatly between samples, and the same was observed for the C:N ratios. The dissolved organic carbon (DOC) concentration was 11.3 gL^–1^ with an origin mostly from plant degraded material, with low bioavailability (bix = 0.681; *fI* = 1.38, and hix = 0.887). Soluble reactive phosphorus (SRP) fluctuated between day and night samples, but not significantly throughout the different sampling days (Table 1). For chlorophyll *a* content (Chl-*a*), a significant difference was found between day and night samples (*p* < 0.001). The pigment composition in relation to the Chl *a* content, analyzed with CHEMTAX, showed that both Euglenophytes and Chlorophytes were the most abundant phototrophic groups in almost all samples (Figure 1A). The relative abundance of almost all phytoplankton groups and particularly Chrysophytes, Cryptophytes, and Cyanobacteria decreased during the night (Figure 1A), which also corresponds to a decrease in their absolute abundance in all night samples (data not shown).

**TABLE 1:** Physicochemical parameters of the pond (mean ± SD of *n* = 4 for *in situ* measured parameters (sampling days), and *n* = 12 for triplicate nutrient analyses per day); numbers in bold indicate significant differences between day and night determined by *t*-test.

**Parameters**	**Day**		**Night**		**Stats**
Temperature [°C] Air	**30.70**	±1.75	**18.43**	±1.28	*P* < 0.001
Water	**24.73**	±0.93	**21.08**	±1.04	*P* < 0.05
PAR [μmol s^–1^ m^–2^] Air	**1999.38**	±73.66	**0.12**	±0.08	*P* < 0.001
Water	**695.25**	±55.67	**0.04**	±0.02	*P* < 0.001
pH	6.63	±0.19	6.39	±0.06	*N*.*S*.
Conductivity [μS cm^–1^]	50.98	±1.25	49.50	±5.78	*N*.*S*.
DO [mg L^–1^]	**3.99**	±1.15	**2.50**	±0.35	*P* < 0.05
Oxygen saturation [%]	**48.09**	±14.28	**28.13**	±4.37	*P* < 0.05
Chl-a [μg L^–1^]	**3.63**	±1.65	**1.46**	±0.99	*P* < 0.001
POC [mg L^–1^]	**1.79**	±0.52	**1.25**	±0.56	*P* < 0.05
PON [mg L^–1^]	0.14	±0.03	0.11	±0.05	*N*.*S*.
C:N	12.69	±1.07	11.83	±1.26	*N*.*S*.
SRP [μg L^–1^]	18.17	±11.02	14.02	±6.17	*N*.*S*.

*N.S. = not significant.*

### Metatranscriptomes

Each sample yielded 14,792,542 to 16,756,988 reads. Even though the samples were poly-A selected for eukaryotic mRNA, low amounts (5–10%) of rRNA were detected and removed with Sortmerna after quality control (QC). The overall assemblies yielded ∼223,000,000–331,000,000 bases per condition and after the annotation, a total of 763,363 and 509,532 transcripts were identified in the day and night metatranscriptomes, respectively.

From the total transcripts assembled, around 40% could be identified as potential proteins once the coding regions were translated, and only ∼15% could be annotated as proteins after performing the protein BLAST against the Swissprot database. From this amount, most of the annotations were assigned to the three ontology categories J (translation, ribosomal structure, and biogenesis), O (post-translational modification, protein turnover, and chaperones), and T (signal transduction mechanisms).

A total of 4693 gene ontology (GO) terms were shared between day and night, 5101 were found to be unique during the day and 4388 were unique for night metatranscriptomes. Among the shared GO terms, cellular components like cytoplasm, nucleus, cytosol, membranes, mitochondrion, chloroplast, endoplasmatic reticulum, and Golgi apparatus were found to be highly abundant, as well as molecular functions like ATP-binding, metal ion-binding and RNA-binding (not shown).

The most abundant GO terms found to be unique for day samples were related to cell defense (defense response to bacteria, GO:0042742), stress mechanisms (downregulation of apoptosis, GO:0043066; heat-shock protein binding, GO:0031072), proliferation and growth (mitotic cell cycle, GO:0000278; nuclear membrane, GO:0031965), and photosynthesis-related processes (photosynthesis, GO:0015979; protein-chromophore linkage, GO:0018298). For the night, the most abundant unique terms were related to protein synthesis and cell growth (meiotic cell cycle, GO:0051321; structural constituent of the cytoskeleton, GO:0005200; ribosome biogenesis, GO:0042254; ribosomal small subunit assembly, GO:0000028, and ribosome binding, GO:0043022) and cell movement (dynein light chain binding, GO:0045503, GO:0051959; ATP-dependant microtubule motor activity, GO:0008569; ciliary basal body, GO:0036064; and cilium movement, GO:0003341, Table 2).

**TABLE 2:** The most abundant GO terms (>2000 annotations per condition and per million, CPM) found to be unique for day and for night samples are shown.

**Ontology**	**GO terms unique day (CPM)**	**GO terms unique night (CPM)**	**GO terms from DE transcripts**
	Kinesin complex (4453)	Ciliary basal body (4841)	Chloroplast thylakoid membrane (5)
	Nuclear membrane (4278)	catalytic step 2 spliceosome (4764)	Cytosol (4)
		lysosomal membrane (4315)	Plasma membrane (4)
		early endosome (4011)	Chloroplast (3)
			Integral component of membrane (3)
Cellular component			Membrane (3)
			Cytoplasm (2)
			Mitochondrial matrix (2)
			Mitochondrion (2)
			Photosystem I (2)
			Plasmodesma (2)
			Proton-transporting ATP synthase complex, catalytic core (2)
	Protein-chromophore linkage (6402)	Ribosomal small subunit assembly (5356)	ATP synthesis coupled proton transport (2)
	Defense response to bacteria (4431)	Ribosome biogenesis (4537)	Photosynthesis (2)
Biological process	Photosynthesis (4311)	Translational elongation (4325)	Photosynthesis, light-harvesting (2)
	Negative regulation of apoptotic process (4241)	Cilium movement (4253)	Response to cytokinin (2)
	Mitotic cell cycle (4066)	Meiotic cell cycle (4155)	Cell-cell adhesion (2)
	Response to cold (4062)		
	Serine-type endopeptidase activity (4837)	Structural constituent of cytoskeleton (5387)	Calcium ion binding (3)
	Protein serine/threonine phosphatase activity (4475)	Dynein light intermediate chain binding (5367)	Electron transfer activity (2)
	Calcium-dependent protein serine/threonine kinase activity (4347)	ATP-dependent microtubule motor activity, minus-end-directed (5166)	GTP binding (2)
Molecular function	Heat shock protein binding (4311)	Dynein light chain binding (5130)	GTPase activity (2)
	Protein domain specific binding (4183)	Ribosome binding (4516)	Oxidoreductase activity (2)
	Metalloendopeptidase activity (4124)		Proton-transporting ATP synthase activity (2)
			Translation elongation factor activity (2)
			Heparin-binding (2)

*The most common GO terms found for all differentially expressed (DE) transcripts during the day (27) are also shown. Ontology is divided into three main categories, and only counts for unique terms were normalized to the total number of annotations per condition and per million (CPM).*

The general taxonomic assignment from the annotated sequences showed that most of the annotations belonged to the phyla Chordata, Streptophyta, Dikarya, and Mycetozoa, and no great variation was observed for day and night samples, except for a small decrease on the abundances at night compared to day (Figure 1B). Instead, when observing at the taxonomic assignment from the differentially expressed transcripts during the day (Figure 1C), the phyla Streptophyta and Chordata were still present, but the rest corresponded to less abundant groups from the general taxonomic assignment: Euglenophyta, Chlorophyta, Apicomplexa, Bacillariophyta, Ciliophora, Nematoda, and Amoebozoa, most of which were confirmed by microscopic observation (data not shown).

### Expression Patterns

**FIGURE 2:**
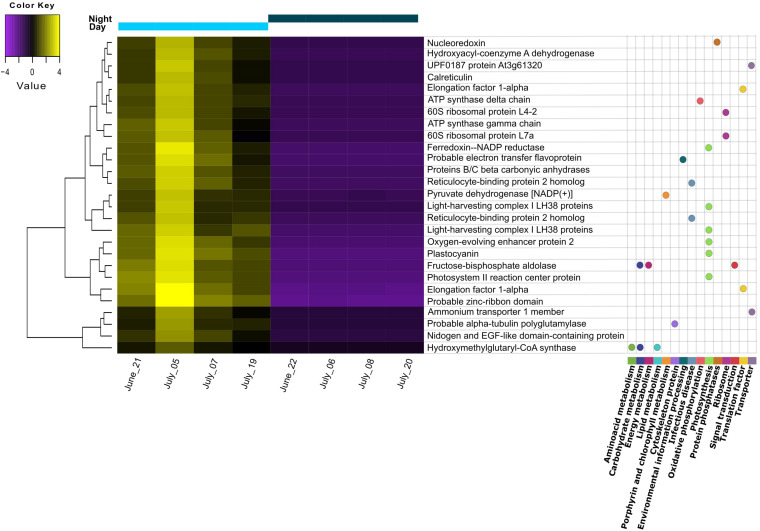
Heatmap of differentially expressed transcripts found to be upregulated during the day. Replicates correspond to different sampling days/nights during summer. Transcripts with annotation are shown with the corresponding pathway from KEGG orthology (KO) assignment. Gene distances were clustered with the Euclidean method. Genes with FDR < 0.01 and at least 2-fold change in expression are shown.

Differential expression (DE) was tested by comparing day and night samples using the initial reference assembly without annotations. We found 45 transcripts to be upregulated during the day. No transcripts were found to be upregulated during the night. From these 45 differentially expressed transcripts, 27 could be functionally annotated. Most of them belonged to the “photosynthesis” category from KEGG Orthology (KO), including light-harvesting complex proteins, photosystem reaction center proteins, oxygen-evolving enhancer proteins, Ferredoxin-NADP reductase, and plastocyanin (Figure 2). The general gene ontology (GO) assignment for the differentially expressed transcripts (Table 2) showed that most of the annotations belonged to parent terms that are expected to be dominant for each of the three GO main categories [cellular component (CC), biological process (BP) and molecular function (MF)], for example, cell and organelle parts including chloroplast, membranes, cytosol, mitochondrion (CC); cellular and metabolic processes like ATP synthesis, photosynthesis, response to cytokinins (BP); catalytic and binding activity like calcium ion-binding, electron transfer activity, GTPase activity (MF), were among the most frequently detected transcripts. From a total of 159 overrepresented (*p* < 0.05) GO terms associated with the DE transcripts, only three GO terms could be identified as enriched (0.05 FDR cutoff). Those were thylakoid (CC), thylakoid membrane (CC), and photosynthetic membrane (CC).

## Discussion

### Expression Patterns

We found that most of the differentially expressed transcripts corresponded to photosynthesis-related machinery and processes. Moreover, the only enriched GO terms from the differentially expressed (DE) transcripts were associated with thylakoids and their membranes. Additionally, chloroplasts were among the most abundant GO terms shared between day and night. A high abundance of chloroplast and photosynthesis-related genes has been also reported in previous summer metatranscriptomic studies, with a higher expression around midday (Ji et al., 2018; Linz et al., 2019). Altogether, this shows the daily rhythmicity of photosynthetic organisms and suggests that, according to what we hypothesized, phototrophic microeukaryotes were more responsive to the day/night cycle than the heterotrophic microeukaryotes in the pond. This response was also consistent over the sampling time, as the replicates corresponded to different dates over a month, but would likely be different during winter, with higher expression of adaptation to cold and low-light mechanisms (Edgar et al., 2016).

Not surprisingly, no upregulation of photosynthesis-related transcripts was detected under dark conditions. Still, these transcripts were not completely absent during the night (Photosystem I, GO:0009522; photosynthesis light-harvesting, GO:0009765, not shown). This has also been recently reported in other studies, where photosystem I (PSI) gene expression would peak at night, and photosystem II (PSII) would have a peak expression at midday (Davenport et al., 2019; Kolody et al., 2019). These studies indicate that either the expression of the photosynthetic machinery would start at night and peak around midday, or that there would be a constitutive expression for some genes/isoforms, and others would be only under/over-expressed if stress conditions are present. The latter has been suggested for some antenna proteins of the light-harvesting complex (Stella, 2016).

In the night dataset, we found unique GO terms referring to cytoskeleton structure, ribosome synthesis, and assembly, indicating the importance of these processes for cell growth during nighttime (Raven, 2013). Furthermore, several GO terms involved in the cellular movement were found to be abundant uniquely at night, like dynein, ATP-dependant microtubule motor activity, ciliary basal body, and cilium movement. Dyneins are ATP-driven protein complexes that are responsible for ciliary and flagellar assembly and movement (Pfister et al., 2006), which could indicate either a higher activity of heterotrophic organisms during the night or simply support the occurrence of cell division processes at this time point.

In general, the expression patterns we found are in agreement with previous metatranscriptomic studies showing that during the day, planktonic cells are actively expressing genes used to obtain energy from light and nutrients, for subsequent cellular growth during the night. At night, genes involved in nucleic acid duplication and ribosomes building up will be activated for protein synthesis and cell division processes, to avoid DNA photodamage caused by UV radiation (Poretsky et al., 2009b; Vila-Costa et al., 2013; Broman et al., 2017; Kolody et al., 2019).

### Transcripts Taxonomic Assignment

From the taxonomic assignment based on the metatranscriptome, an exclusively marine group (Echinodermata), and also Arthropoda, Chordata, Cnidaria, Mollusca, Mycetozoa, and Platyhelminthes were assigned to our pond community. As we had deliberately excluded most metazoans by pre-filtering our water samples (<30 μm), the occurrence of these taxa was thus unexpected. Nevertheless, it must be taken into account that this taxonomic assignment is based on functional annotations rather than on the commonly used 18S rRNA gene sequences, as the poly-A transcripts selection we used discriminates against ribosomal and prokaryotic RNA. Still, we can infer from these general functional taxonomy data that the annotations belonging to the above-mentioned groups are most probably not exclusive but rather shared functions across taxa, normally biased by the much more comprehensive data available for model organisms (Shakya et al., 2019). The phototrophic fraction was not the most abundant in the pond, as reflected in the metatranscriptomes taxonomical composition. This was also supported by a weakly positive correlation between POC and chlorophyll *a* (*r*^2^ = 0.5538), indicating that most of the POC in the pond did not belong to phototrophic organisms. Based on the CHEMTAX assignment from the pigment analyses, we were able to classify the phototrophic community into the major phytoplankton divisions. It should be noted that this CHEMTAX-based assignment also includes the non-eukaryotic cyanobacteria in the analysis because their marker pigments (echinenone and zeaxanthin) were detected. Excluding this non-eukaryotic contribution to total Chl-*a* would have been redistributed to the eukaryotic groups, which would have resulted in a biased community composition. Generally, we observed a decrease in most phototrophic groups at night, which could indicate that these groups were grazed by heterotrophs. An alternative explanation is that they were able to perform vertical or horizontal migrations, as it has been shown for several phytoplankton groups that take advantage of surface light and hypolimnetic nutrients through the diurnal cycle (Roenneberg and Mittag, 1996; Shikata et al., 2015). The most abundant phyla in our pond were Chlorophyta and Euglenophyta. The high abundance of Euglenophytes and their differential expression response to the diel cycle suggests that this diverse group of organisms, including mixotrophs, might be the most successful phytoplanktonic group in the pond. This success is perhaps due to their flagella and stigma (“eye spot”), facilitating movement toward best light conditions (phototaxis), but also to their ability to form cysts when environmental conditions deteriorate (Schwartzbach and Shigeoka, 2017).

### Environmental Variables

We expected to find diel rhythmicity for the POC and PON content, due to the increasing biomass of primary producers during the day and the nitrogen fixation that typically occurs at night (reviewed in Berman-Frank et al., 2003) but we only found a diel fluctuation on POC. The C:N ratios did not vary significantly between day and night, but the values (∼12) were twice the Redfield ratio (C:N 6.7, Redfield, 1958), which could indicate a moderate nitrogen deficiency in the pond’s plankton (Hecky et al., 1993).

Another possible limiting factor for the phototrophic fraction could be the incident light, as direct light was limited by tree shading in the surroundings and floating waterlily (*Nymphaea* sp.) leaves on part of the pond’s surface. Macrophytes such as waterlilies not only limit light for the pond phytoplankton but could additionally be competitors for inorganic nutrients (Bolpagni et al., 2014). The plant material surrounding the pond (allochthonous) explained the high DOC concentrations determined for our pond (11.3 g L^–1^) which are similar to what is found in peatlands (Graeber et al., 2012). Allochthonous DOC is often contributing to the phosphorus input into aquatic systems (Nürnberg and Shaw, 1998). We measured high soluble reactive phosphorus concentrations in the pond (∼15–20 μg L^–1^), indicating that phosphorus availability was not limiting primary production. On the contrary, the high DOC levels from plant origin in the pond could be negatively affecting primary production in the system due to the decreased light incidence, as shown for high DOC-colored lakes (Klug, 2002). Such a simultaneous light and nutrient limitation of the phototrophs would favor the heterotrophic microbial community.

### From Functions to the Environment

Our results suggest that the microbial primary producers in the pond were not limited by phosphorus, but rather by nitrogen and/or light availability. The phytoplankton C:N for day and night samples pointed to a deficiency of N in the pond, suggesting that strategies to improve N uptake are needed. One of these strategies is to increase transporter proteins for nutrient uptake, and we can see in our results from DE transcripts and functions that the only identified transporter upregulated during the day is an ammonium transporter. Similarly, in cases were phosphorus is deficient, a general upregulation of different phosphate transporters should be found, as described in previous metatranscriptome studies (Vila-Costa et al., 2013; Zhang et al., 2019), we did not identify this for the pond metatranscriptomes, supporting that P was not limiting in the system.

The phototrophic fraction was the most responsive to high light during midday, which was initially expected as a consequence of a higher phytoplankton abundance during summer with concomitant higher temperature and light availabilities (Schwaderer et al., 2011; Edwards et al., 2016). However, since light availability in the pond was limited not only in time but also in intensity, as a consequence of the high DOC in the water, primary production was reduced. Consequently, the phototrophic fraction was more responsive than the heterotrophic fraction, as they needed acclimation mechanisms in the light-limited system. These mechanisms might involve the enhancement of light-harvesting for energy acquisition and the regulation of stress responses.

**FIGURE 3:**
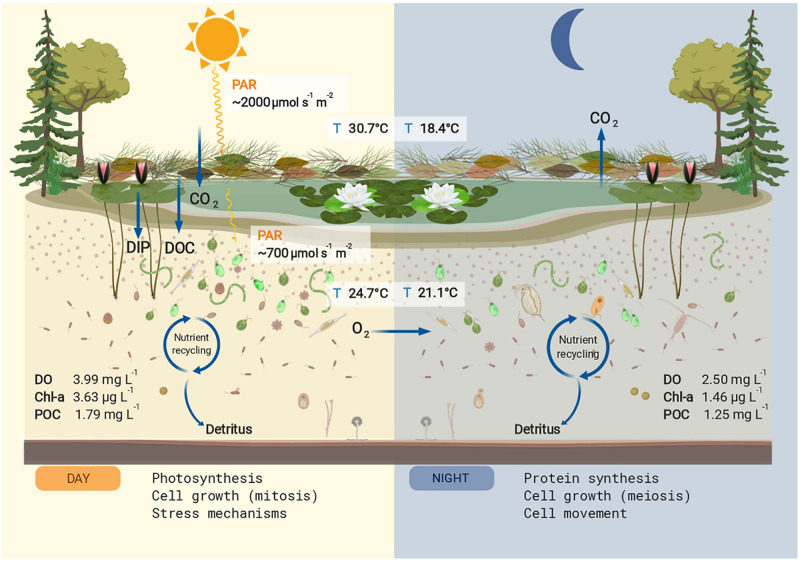
Conceptual summary of the studied pond. The mean values for physicochemical parameters that differed significantly between day and night are shown. Nutrient input for dissolved organic carbon (DOC) and dissolved inorganic phosphorus (DIP) are shown in the left panel. During the day the main cellular process upregulated in the microeukaryotic community was photosynthesis, increasing particulate organic carbon (POC), chlorophyll *a* (Chl *a*), and dissolved oxygen (DO). The produced and stored energy is used in part for cell growth and stress mechanisms, also upregulated processes during the day. During the night, the oxygen produced via photosynthesis is used for respiration, decreasing the DO concentrations. The main cellular process upregulated at night was protein synthesis, a high energy-consuming task, as well as cell growth and cell movement. The POC and Chl *a* values decreased at night presumably by grazing effect. This figure was created with BioRender.

Accordingly, we found a high expression of stress response mechanisms like heat-shock proteins (HSP) during the day, indicating cellular stress for the pond’s microeukaryotes. HSP are synthesized to protect against protein denaturation when environmental stressors such as high temperature, light, UV, are present (Guo et al., 2015; Sathasivam and Ki, 2019). Apart from light, the temperature was another parameter with pronounced diurnal fluctuations. Considering that light intensity was rather limited, the high expression of HSP might point to temperature stress rather than high light stress. Interestingly, as shown on the heatmap, the highest expression among replicate days for all DE transcripts was observed for July 5th, when the midday water temperature was the lowest (below 24°C) and PAR values were similar to the other days. We also found a high expression for other stress-related responses: defense response to bacteria and downregulation of apoptosis are GO terms that might indicate defense mechanisms to heterotrophic bacterial infection, although we cannot attribute this stress response to a specific group of organisms in the pond community. An additional indication of stress to temperature and nutrient limitation is the presence of meiosis among the most abundant GO terms during the night, as sexual reproduction in microalgal cells is known to be induced by suboptimal conditions for growth (Borowitzka, 2018, and references therein). The summary of our findings on the pond ecosystem during summer is shown in Figure 3.

Overall, for our metatranscriptomics study, we showed that by combining this gene expression approach with other methods such as pigment analyses, and environmental parameters, a better understanding of the system could be obtained. However, the functions identified in our results cannot be attributed to specific organisms, and rather point out to responses from a part of the pond community. To overcome this limitation other approaches such as single-cell transcriptomics should be used, where a higher diversity of the microeukaryotic fraction and specific dynamic interactions can be identified (Ku and Sebé-Pedrós, 2019; Sieracki et al., 2019). We also acknowledge the importance of considering representative time points, as the metabolic processes of a community can change not only at different times of a day but also daily. The results of our study represent the processes carried by a specific fraction of the pond community during the summer, and should therefore not be extrapolated to other seasons where the observed daily expression patterns are likely to differ. To address further questions on the small pond ecosystem in our study, preferably even more parameters and variables should be considered to fully explain the observed expression patterns. This might ultimately help to link the metabolic processes to specific taxonomic groups and give a broader view of the different trophic levels in the pond. We are aware of the importance of, for example, the measurement of particulate phosphorus as it is essential for the calculation of the C:N:P stoichiometry from primary producers and to unveil their specific nutrient requirements and limitations. Also, photosynthesis rates and photoinhibition would have been interesting to determine primary productivity and light/temperature stress responses in the pond, respectively. Likewise, considering the identification of other size fractions would contribute to better understand the trophic interactions in the pond community.

## Conclusion

Our study revealed that the phototrophic pond microeukaryotes were the less abundant but the most responsive fraction to diurnal fluctuations. This was indicated by the strong phototrophic contribution to the differentially expressed transcripts, the most abundant gene ontology terms shared between day and night samples, and the GO terms found to be unique for the daytime samples. In all cases, photosynthesis-related annotations/processes were prevailing. Furthermore, the expression patterns observed for day and night samples were consistent over the sampling period and were confirmed to be similar to some previous studies in larger aquatic ecosystems, remarking that the diurnal cycles affect the pond community in the same way.

Moreover, combining the metatranscriptomics with pigment analyses allowed us, together with the environmental data, to identify the phototrophic composition on the whole microeukaryotic community and to better understand the main metabolic processes in response to limiting factors for primary production in a small aquatic ecosystem.

## Data Availability Statement

The data from gene expression and gene ontology analyses is available on the Github repository https://github.com/sjtf89/Cologne_pond. The raw sequences are deposited in the sequence read archive (SRA) database from NCBI under the BioProject PRJNA596111.

## Author Contributions

Both authors conceived the study and wrote the manuscript. ST-F carried out samplings and processing and analyzed the metatranscriptomes.

## Conflict of Interest

The authors declare that the research was conducted in the absence of any commercial or financial relationships that could be construed as a potential conflict of interest.

## Footnotes

1 http://transdecoder.github.io
